# Screening assays for primary haemophagocytic lymphohistiocytosis in children presenting with suspected macrophage activation syndrome

**DOI:** 10.1186/s12969-015-0043-7

**Published:** 2015-11-16

**Authors:** Mary Cruikshank, Parameswaran Anoop, Olga Nikolajeva, Anupama Rao, Kanchan Rao, Kimberly Gilmour, Despina Eleftheriou, Paul A Brogan

**Affiliations:** Departments of Rheumatology, Great Ormond Street Hospital NHS Foundation Trust, London, UK; Departments of Haematology, Great Ormond Street Hospital NHS Foundation Trust, London, UK; Departments of Bone Marrow Transplantation, Great Ormond Street Hospital NHS Foundation Trust, London, UK; Departments of Immunology, Great Ormond Street Hospital NHS Foundation Trust, London, UK; Institute of Child Health, University College London, London, UK; Arthritis Research UK Centre for Adolescent Rheumatology, London, UK

**Keywords:** Haemophagocytic lymphohistiocytosis, Macrophage activation syndrome, Protein screening assays, CD107a granule release assay, Children

## Abstract

**Background:**

Primary haemophagocytic lymphohistiocytosis (HLH) screening assays are increasingly being performed in patients presenting with macrophage activation syndrome (MAS). The objective of this study was to describe their diagnostic and prognostic relevance in children who had presented to paediatric rheumatology and had undergone investigative work up for MAS.

**Methods:**

Data was obtained retrospectively from an existing protein screening assay database and patient records. Assays included: intracellular expression of perforin in CD56+ Natural Killer (NK) cells; CD107a Granule Release Assay (GRA) in response to PHA in NK cells, or anti-CD3 stimulation of CD8 lymphocytes; in males Signal Lymphocyte Activating Molecule Associated Protein (SAP), and X-linked Inhibitor of Apoptosis Protein (XIAP) expression. All assays, requested by paediatric rheumatology, of children who had undergone investigative work up for MAS over a 5-year period (2007–2011) were included.

**Results:**

Twenty-one patients (15 female), median age 6.5 years (range 0.6–16) with follow-up of 16 months (range 1–51), were retrospectively identified. At presentation, 3/21 (14 %) fulfilled HLH-2004 diagnostic criteria. At least one screening test result was available for all 21 patients; 7/21 (33 %) had at least one persistent screening test abnormality. Of this group 4/7 (57 %) died or required haematopoietic stem cell transplantation (HSCT), compared to 1/14 (7 %) with no screening test abnormality (*p* = 0.025). 3/21 (14 %) ultimately had a diagnosis of primary HLH (two confirmed genetically; XIAP, familial HLH type 3, and one confirmed clinically). Of the six patients with abnormal GRA 5/6 had negative routine genetic results.

**Conclusions:**

Screening for primary HLH is warranted for children whose first rheumatological presentation is with MAS, since overall 14 % had an eventual diagnosis of primary HLH. A persistently abnormal GRA in patients presenting with MAS defines a high-risk group with poor outcome (mortality or HSCT), possibly due to as yet unidentified genetic cause.

**Electronic supplementary material:**

The online version of this article (doi:10.1186/s12969-015-0043-7) contains supplementary material, which is available to authorized users.

## Background

**Fig. 1:**
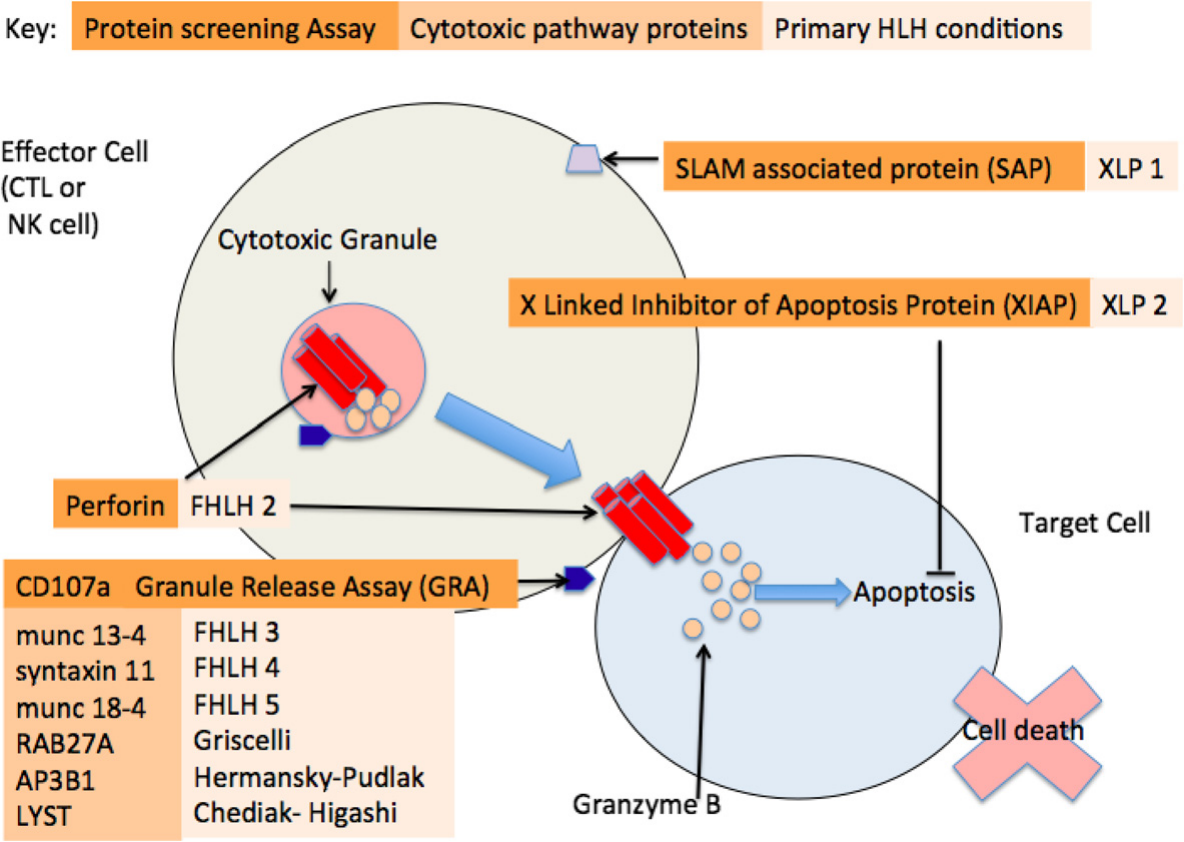
Proteins screened in relation to pathways involved in pathogenesis of primary haemophagocytic lymphohistiocytosis (HLH). Cytotoxic T lymphocytes and natural killer cells contain specialized secretory lysosomes - cytotoxic granules, which upon contact with a target-cell degranulate. These granules contain perforin, a membrane-disrupting protein that facilitates the delivery of granzymes (proteases) that initiate apoptotic death in target-cells. If perforin is deficient, it is suggestive of a diagnosis of Familial HLH type 2. To check the degranulation process, we can artificially activate NK and CTL cells in the laboratory and measure using flow cytometry, a cell surface protein, CD107a (Granule Release Assay (GRA). If abnormal, it is an indirect marker of a host of proteins which may be involved in the degranulation process - for example in trafficking, docking, priming for exocytosis, or membrane fusion. An abnormal GRA may be suggestive of a diagnosis of familial HLH 3–5 or Griscelli, Chediak-Higashi or Hermansky-Pudlak. In boys there are two X-Linked conditions, which result in protein deficiencies which can be screened for; SLAM – Associated Protein (SAP) which is important in the killing of EBV infected cells – if deficient – suggestive of X linked Lymphoproliferative Disease ((XLP) type 1, and X Linked Inhibitor of Apoptosis Protein (XIAP) which if deficient – suggestive of XLP type 2. *XLP* X linked lymphoproliferative disease type 1, *XLP2* X linked lymphoproliferative disease type 2, *FHLH* familial haemophagocytic lymphohistiocytosis, *CTL* cytotoxic T lymphocyte, *NK* natural killer

Haemophagocytic lymphohistiocytosis (HLH) is a life-threatening condition characterised by excessive activation of T cells and macrophages and extreme uncontrolled inflammation driven by γ-interferon and other pro-inflammatory cytokines [1]. Traditionally HLH is classified into primary (genetic/familial) or secondary forms [2]. There is an increasing understanding of the genetic and molecular basis of many primary forms of HLH, summarised in Fig. 1 [3–10]. Secondary HLH may occur in patients with infections such as Epstein Barr Virus; haematological malignancies; metabolic conditions; and a range of autoimmune and other inflammatory conditions [2, 11]. When HLH is secondary to these latter rheumatological conditions, it is usually referred to as macrophage activation syndrome (MAS) [12]. This can be a severe and potentially fatal complication if not recognised early and treated aggressively.

In children, distinguishing between primary HLH and MAS can be challenging particularly when the first presentation of a rheumatological disorder is with MAS. Whilst the clinical features (including in some cases infectious triggers) may be identical for both primary HLH and MAS and in some instances rescue therapies the same, ultimately the treatments for these diseases differ significantly. For example, paediatric patients with primary HLH require rescue therapy with high-dose corticosteroids, ciclosporin, etoposide, and ultimately allogeneic haematopoietic stem cell transplantation (HSCT) [13]. Whilst these treatment approaches are required in a minority of patients with MAS, usually high-dose corticosteroids, ciclosporin and treatment of the underlying inflammatory disorder driving the MAS suffices [12]. Thus, distinguishing between primary HLH and MAS is of utmost clinical importance in order to instigate appropriate rescue therapy, provide prognostic information, plan for allogeneic HSCT where required, and to provide appropriate genetic counselling [14].

Currently the diagnosis of primary HLH is based on a set of clinical and laboratory parameters that have been developed and modified for use in therapeutic clinical trials [15]. This diagnosis requires either a molecular diagnosis (genetic confirmation), or 5/8 clinical criteria (Table 1) being met. However, the specificity of these criteria, particularly in differentiating primary from secondary HLH, is not well established. In addition, obtaining a confirmatory definitive molecular diagnosis may take several weeks or even months, and other genetic forms of HLH are yet to be described [16]. Hence genetic confirmation of primary HLH is of limited value to guide clinical management in the acute setting [13]. Diagnostic guidelines for MAS complicating sJIA and SLE exist [17–20]. Although these guidelines may be helpful, they have some limitations [21–23] including the fact that if MAS is the main first presenting feature, the patients’ rheumatological diagnosis may still be unclear.

**Table 1:** Haemophagocytic Lymphohistiocytosis (HLH) 2004 clinical trial B diagnostic (clinical) criteria from 21 patients who had investigative work up for macrophage activation syndrome at presentation and underwent protein-based and degranulation screening tests for primary HLH from a single United Kingdom Paediatric Rheumatology centre

	Number of patients (%)	Number of patients with information available
HLH diagnosis: B Criteria		
Fever	19 (90)	21
Splenomegaly	9 (47)	19
Cytopenia (affected ≥2 of 3 lineages)	5 (24)	21
Haemoglobin <9 g/dl	11 (52)	
(Infants <4 weeks: Haemoglobin <10 g/dl)		
Platelets <100 × 10^9^/l	7 (33)	
Neutrophils <1 × 10^9^/l	2 (1)	
Hypertriglyceridaemia and/or hypofibrinogenaemia:		
Fasting triglycerides ≥3 mmol/l	8 (53)	15
Fibrinogen ≤1.5 g/l	1 (0.1)	20
Haemophagocytosis in bone marrow or spleen or lymph nodes & no evidence of malignancy	7 (41)	17
Low or absent natural killer cell activity	Not tested	
Ferritin ≥500 μg/l	14 (74)	19
^a^Soluble CD25 ≥2400 U/ml	4 (100)	4

^a^Only four patients had soluble CD25 levels tested. All were ≥2400 U/ml based on the original description of raised levels. It is acknowledged that >2SD from the mean is thought to be more meaningful

Increased understanding of the cell cytotoxic pathways involved in the pathogenesis of primary HLH has led to the development of a number of assays which may be used to rapidly identify patients with primary HLH [24]. These include: flow cytometry–based protein screening assays that quantify perforin; SLAM-associated protein (SAP); X-linked inhibitor of apoptosis (XIAP) expression; and surface expression of CD107a, a surface lysosomal protein in cytotoxic granules used to quantify degranulation in the cytotoxic cellular pathway (Fig. 1). These screening tests have a quick return of results (within 48 h) and can therefore help guide acute management since it is generally assumed that abnormal results are due to underlying genetic cause and hence indicate primary forms of HLH [24]. However, given the overlapping clinical features of primary HLH and MAS, similar cytotoxic cellular pathways may also be involved in the pathogenesis of MAS [25]. For instance natural killer (NK) cell dysfunction is recognised in MAS complicating systemic Juvenile Idiopathic Arthritis (sJIA) [26, 27]. Reduced perforin expression has been observed in patients with sJIA [28] and perforin gene mutations and munc 13-4 polymorphisms have been linked to risk of MAS in sJIA [29, 30]. However, the performance of the aforementioned newer functional protein screening assays in the evaluation of patients with MAS has not yet been established.

We hypothesized that screening assays for primary HLH could be relevant to children with MAS either by: 1. identifying those with potential primary genetic HLH; and/or 2. defining a hitherto unrecognised subgroup of MAS with poor prognosis. The aims of this study were therefore to report the performance of these protein screening assays in a cohort of children referred to our tertiary paediatric rheumatology centre who underwent investigative work up for MAS due to suspected but as yet undetermined rheumatological cause. Specifically, we wished to study any potential association between the presence of any abnormal screening assay result, final diagnosis, and outcomes including mortality or HSCT.

## Methods

### Study population

This was a 5-year retrospective review of children referred to paediatric rheumatology at Great Ormond Street Hospital National Health Service Foundation Trust between 1^st^ January 2007 (when the CD107a assay became routinely available at our institution) and December 31^st^ 2011. We included all patients who underwent protein screening assays during this time period for the work up of MAS due to suspected but as yet undefined rheumatological cause. Patients were identified through a search of hospital based clinical and laboratory databases. Data collected included: sex, ethnicity, age at time of screening, disease duration, length of follow up, presenting clinical features, and the frequency of HLH 2004 clinical diagnostic criteria parameters at time of screening. Outcomes included final diagnosis, mortality or HSCT. All blood samples were collected during the acute presentation with active disease. Local research and development approval was obtained for retrospective case review (reference 12RUO1), and exemption from ethics approval, under the Governance Arrangements for Research Ethics Committee 2011, was granted.

### Screening assays

Standard operating procedures for the granule release assay and detection of perforin, XIAP and SAP are included as a supplement [Additional file 1]. Cells were permeabilised and stained as per Bryceson et al. [31]. In brief whole blood samples (5–10 mL) were collected in EDTA-containing vials. One healthy control sample was processed in parallel to each patient sample.

For the granule release assay, peripheral blood mononuclear cells were separated from whole blood and stimulated overnight with IL-2. The cells were then stimulated with PHA or anti-CD3 antibody in the presence of FITC anti-CD107a antibody for 2 h, then stained with cell surface markers and analysed by flow cytometry. An abnormal GRA result is when the increase in %CD107a between stimulated and unstimulated samples was <1.5 % for cytotoxic T cells after anti CD3 stimulation and/or <15 % for NK cells after PHA stimulation [32].

Whole blood intracellular FACs for SAP, XIAP and perforin was performed by flow cytometry. Perforin is abnormal if <50 % expression and/or fails to have the brightest cells reach Mean Fluorescein Intensity of 10^3^. SAP and XIAP are abnormal if <50 % of cells express SAP/XIAP.

### Genetic analyses

Sanger sequencing of exons and exon/intron boundaries was undertaken on all patients with abnormal protein expression or degranulation. Primers and sequencing conditions are available on request.

### Statistical analyses

Fisher Exact Test was used to compare the outcome between patient groups categorised based on the presence of an abnormal functional assay test. Continuous variables were presented as median and range, and categorical data as percentages and proportions. SPSS version 18 was used for statistical analysis. *P* values of <0.05 were considered significant.

## Results

### Patient demographics

Twenty-one patients (15 female) underwent screening tests. Ethnicity included white British (11), Asian/Asian British (3), black African/black British (4), Arab (1), white other (2). Median age at time of screening was 6.5 years (range 0.6–16.0) and median disease duration of 67 days (range 24 days– 6 years). One patient (an outlier) who had disease duration of 6 years was managed at another hospital prior to having been referred to the paediatric rheumatology service. Median follow-up was 16 months (range 1–51 months).

### Clinical features and laboratory tests

Of the results available, only 3/21 (14 %) fulfilled a minimum of 5/8 of the HLH -2004 (B) diagnostic clinical criteria (Table 1). At presentation 19/21 (90 %) had fever; 12/20 (60 %) had hepatomegaly; 11/21 (52 %) had arthritis; 9/19 (47 %) had splenomegaly; and 6/20 (30 %) had evidence of central nervous system involvement (defined as irritability, disorientation, headache, seizure or coma). The median ferritin was 2531 (range 38–22076) μg/l; median ESR 23 (range 3–160) mm/h; and median CRP 39 (range <5–187) mg/l. Table 2 summarises the clinical and laboratory features for the patients with abnormal screening results. Summary data set for each patient is available [Additional file 2].

**Table 2:** Clinical and laboratory features of 7 patients referred with suspected macrophage activation syndrome who had abnormal screening results (GRA and XIAP)

Patient	3	4	5	6	7	8	18
Sex	f	f	f	m	f	f	m
HLH 2004 B clinical criteria							
Fever	yes	yes	yes	yes	no	yes	yes
Splenomegaly	yes	/	yes	no	no	no	yes
Cytopenia at least 2/3	yes	no	yes	yes	yes	no	yes
Hb <90	yes	yes	yes	yes	yes	yes	no
Plt <100	no	no	yes	yes	yes	no	yes
Neut <1	yes	no	no	no	no	no	yes
Hypertriglyceridemia &/or hypofibrinogen	yes	yes	/	no	/	yes	/
Triglycerides >265	yes	yes	/	no	/	yes	/
Fibrinogen <1.5	no	no	no	no	no	no	no
Haemophagocytosis in bone marrow, spleen or lymph nodes	no	no	/	no	yes	yes	/
Low or absent NK cell activity	/	/	/	/	/	/	/
Ferritin >500 ng/ml	yes	yes	yes	yes	yes	yes	no
Soluble CD25 >2400units/ml	yes	/	/	/	/	/	/
Number of HLH B clinical (2004)	6	3	4	3	3	4	3
Ferritin ng/ml	21065	1679	523	6759	2801	22076	38
ALT IU/L	17	17	12	453	6	354	102
Arthritis	yes	no	no	yes	yes	yes	yes
ESR mm/h	100	10	78	20	18	120	6
CRP mg/L	48	74	60	/	51	87	6
Screening tests							
GRA	abnormal	abnormal	abnormal	abnormal	abnormal	abnormal	normal
SAP	/	/	/	normal	/	/	normal
XIAP	/	/	/	normal	**/**	/	absent
PERFORIN	normal	normal	normal	normal	/	normal	normal
Genetics sent	yes	yes	yes	yes	yes	yes	yes
Genetics	no mutation found	c.2335G>A/wt and possible deletion/splicing defect	no mutation found	no mutation found	no mutation found	no mutation found	c.1082C>G (p.Ser361X)
Diagnosis	sJIA	primary HLH (FHLH type 3)	primary HLH	overlap syndrome (JDM//SLE/sJIA)	SJIA	SJIA	primary HLH (XLP2)
HSCT	no	no	no	no	yes	no	yes
Survival	alive	deceased	deceased	alive	alive	alive	alive

/= not done/no data available

*HLH* haemophagocytic lymphohistiocytosis, *Hb* haemoglobin, *Plt* platelets, *Neut* neutrophils, *NK* natural killer, *AST* aspartate aminotransferase, *WCC* white cell count, *CNS* central nervous system, *SoJIA* systemic onset juvenile idiopathic arthritis, *ALT* alanine transaminase, *ESR* erythrocyte sedimentation rate, *CRP* C reactive protein, *GRA* granule release assay, *SAP* SLAM associated protein, *XIAP* X linked inhibitor of apoptosis protein, *HSCT* haematopoietic stem cell transplantation, *JDM* juvenile dermatomyositis, *SLE* systemic lupus erythematosus, *XLP2* X linked lymphoproliferative syndrome type 2

### Protein and degranulation assay results

At least one screening test result was available for all 21 patients. Nineteen patients had the CD107a GRA performed; for two patients the assays were technically inadequate to interpret. A total of 6/17 (35 %) had a persistently abnormal GRA. A persistently abnormal GRA was defined as a minimum of two abnormal GRA, as per routine clinical practice). The median time between first and second abnormal GRA was 27.5 days (range 4 – 276 days). One boy had absent XIAP expression. Nineteen patients had perforin screened, and five male patients were screened for SAP expression; all had normal perforin and SAP results respectively.

### Final diagnoses

All seven patients with abnormal screening assays (six with abnormal GRA, and one with absent expression of XIAP) went on to have genetic testing for known mutations associated with primary HLH. One patient with absent expression of XIAP had subsequent genetic confirmation of XLP type 2 (c.1082C>G (p.Ser361X)). Only 1/6 patients with an abnormal GRA had positive genetics (heterozygous UNC13 mutation as well as a second heterozygous sequence variant, suggesting the diagnosis of Familial HLH type 3); the remaining 5/6 patients with abnormal GRA had no identifiable mutations in munc or syntaxin genes. Their final diagnoses were: sJIA with MAS (*n* = 3); primary HLH but without genetic confirmation (*n* = 1); and overlap syndrome with features of juvenile dermatomyositis, systemic lupus erythematosus and sJIA (*n* = 1). The final diagnoses of the 11/17 patients with normal GRA screening test abnormality are provided in Table 3.

**Table 3:** Outcome - mortality or haematopoietic stem cell transplantation (HSCT) in relation to presence of protein based and degranulation screening abnormalities in 21 patients with referred with suspected macrophage activation syndrome

	Mortality or HSCT	Alive and no HSCT	Total	*P* value
**Abnormal screening test**	4	3	7	
CD107a GRA (persistently abnormal)	Primary HLH *n* = 2 (1 UNC13D variant)	sJIA *n* = 2		
	sJIA *n* = 1	Overlap syndrome (JDM/SLE/sJIA) *n* = 1		
Absent XIAP	Primary HLH (XLP2) *n* = 1	*n* = 0		
**Normal screening test**	1	13	14	0.0253
Normal:		sJIA *n* = 4		
CD107a GRA	sJIA *n* = 1	SLE *n* = 1		
Perforin		Streptococcal infection *n* = 1		
XIAP		Pancreatitis and SIRS *n* = 1		
SAP		Unclassified immunodeficiency *n* = 1		
		Langerhans Cell Histiocytosis *n* = 1		
		No clear diagnosis *n* = 4		

Final diagnoses listed. Children with abnormal screening test results were more likely to die or undergo HSCT. *p* <0.05 by Fisher Exact Test was considered significant

*HLH* haemophagocytic lymphohistiocytosis, *GRA* granule release assay, *sJIA* systemic juvenile idiopathic arthritis, *JDM* juvenile dermatomyositis, *SLE* systemic lupus erythematosus, *XIAP* X linked inhibitor of apoptosis protein, *XLP2* X linked lymphoproliferative syndrome type 2, *SAP* SLAM associated protein, *SIRS* systemic inflammatory response syndrome

### Outcomes

Of the group of children with any persistent screening test (GRA and XIAP) abnormality, 4/7 (57 %) either died (2/7), or required HSCT (2/7), compared to 7 % with no screening abnormality (none died, and 1/14 underwent HSCT); *p* = 0.025; Table 3. When data was analysed specifically comparing outcome between abnormal and normal GRA it was not found to be statistically significant.

## Discussion

In this study we report the use of protein-based and degranulation screening assays for primary HLH in a cohort of children referred to our tertiary paediatric rheumatology centre with MAS due to suspected but as yet undetermined rheumatological cause. We established that these functional tests can direct genetic analyses and may allow a rapid and reliable classification of patients, and are important prognostically. The presence of persistently abnormal screening tests, defined a high risk group with poor outcome (mortality or HSCT in 4/7 cases, 57 %). Although our data did not show a statistical difference in outcome for patients when the data was specifically analysed comparing those who had abnormal and normal GRA tests, our numbers are small and it is important still to highlight that 3/6 patients (50 %) who had an abnormal GRA either died or required HSCT. Overall 3/21 (14 %) of our patients who had protein-based and degranulation screening tests were diagnosed with primary HLH (two confirmed genetically, one clinical diagnosis); novel molecular defects may be implicated in the pathogenesis of HLH in the remaining cases with abnormal GRA but negative routine genetic results. To date, stratification of the mortality risk and/or the need for urgent consideration of HSCT in patients presenting with MAS has not been possible. Our findings, albeit preliminary and retrospective, suggest for the first time that a persistently abnormal CD107a degranulation screening assay may identify patients presenting with MAS with poorer outcomes. Further prospective multicentre studies are now needed to validate this observation.

Our study focused on children in whom MAS was the presenting feature and the remaining clinical manifestations were initially atypical for an established underlying inflammatory disease. As primary HLH and MAS overlap clinically, we suggest that rapid screening for primary HLH in these patients is warranted; however, clinicians could consider these investigations for children with MAS even with an established underlying rheumatological disorder, particularly for those cases not responding to high-dose corticosteroids and ciclosporin. Abnormal screening test findings could also define a high risk group in that context, and this remains an area worthy of future study.

Notably, three patients with sJIA and one patient with overlap syndrome (with features of juvenile dermatomyositis, systemic lupus erythematosus and sJIA) had a persistently abnormal CD107a test with negative genetic tests (Table 3), suggesting that this assay is not entirely specific for primary forms of HLH, and that this pathway could be involved in the pathogenesis of MAS in some patients. A recent study using whole-exome sequencing in patients with systemic JIA and MAS identified rare protein-altering variants in known HLH-associated genes as well as in new candidate genes [33]. This may be of relevance for our cohort. Importantly, we have not yet examined the effect of immunosuppressive treatment on this assay on this cohort of patients. However, in patients with primary HLH treated with HLH-94 and HLH-2004 treatment protocols, immunosuppressive therapy does not significantly affect the degranulation assay result [31]. Previous studies have suggested temporary defects of NK cell perforin expression in children with active sJIA that normalises with successful treatment [16]. However, in our cohort of patients, perforin expression was normal.

Our study is limited by all the confounding factors associated with any retrospective case series, and we acknowledge the small size and heterogeneity of this cohort. In addition, there is a possibility that referral bias resulted in us receiving the most severe cases of secondary HLH/MAS. It is noted that perforin and SAP assays were all normal in this cohort. Given the small cohort size and rarity of primary HLH conditions it would be incorrect to conclude from these findings that they are not clinically helpful. As previously mentioned, the longitudinal effect of immunosuppressive treatment was not assessed, and requires further investigation.

## Conclusions

This study provides support that screening for primary HLH is warranted for children whose first rheumatological presentation is with MAS, since overall 14 % had an eventual diagnosis of primary HLH. A persistently abnormal GRA in patients presenting with MAS defines a high-risk group with poor outcome (mortality or HSCT), possibly due to as yet unidentified genetic cause. Protein-based and degranulation screening assays may therefore facilitate rapid identification of high risk groups of patients referred to paediatric rheumatology with MAS; we suggest these tests may be able to help the clinician in those first few days to reach a diagnosis of either primary HLH or MAS, and therefore may be able to guide treatment decisions and improve prognosis for this potentially fatal complication.

## Additional files

Additional file 1:
**Standard Operating procedures.** Standard operating procedures for the granule release assay, and detection of perforin, XIAP and SAP. (DOCX 1303 kb) Additional file 2:
**Data set summary.** Clinical information from 21 patients who underwent screening for suspected MAS clinical information. (DOCX 317 kb)

## Abbreviations

GRA: granule release assay
HLH: haemophagocytic lymphohistiocytosis
HSCT: haematopoietic stem cell transplantation
JIA: juvenile idiopathic arthritis
MAS: macrophage activation syndrome
NK: natural killer
SAP: signal lymphocyte activating molecule associated protein
sJIA: systemic juvenile idiopathic arthritis
XIAP: X-linked inhibitor of apoptosis protein
