# Supplementary material for: Screening assays for primary haemophagocytic lymphohistiocytosis in children presenting with suspected macrophage activation syndrome
Source: Pediatr Rheumatol Online J. 2015 Nov 16;13:48. doi: 10.1186/s12969-015-0043-7 (PMC4647814; doi:10.1186/s12969-015-0043-7)
Supplement: Additional file 1: — Standard Operating procedures. Standard operating procedures for the granule release assay, and detection of perforin, XIAP and SAP. (DOCX 1303 kb) [file 12969_2015_43_MOESM1_ESM.docx]

**Supplemental Methods**

**Standard Operating Procedures for Granule Release Assay, Detection of Perforin, SAP and XIAP by FACS**

Department of Immunology, Camelia Botnar Laboratory, Great Ormond Street Hospital, London

**1. Granule Release Assay**

**A. EQUIPMENT**

Class II safety cabinet

37°C CO_2_ incubator

Centrifuges

Universal rack

Gilson pipettes

Eppendorf repeater

Pipette aid

Discard container

Tube racks

FACSCalibur

Haemocytometer

**B. REAGENTS**

**Reagent Supplier Cat. No. Hazard**

*RPMI 1640 Invitrogen 52400-025. R3

*Gentamycin Pharmacy N/A G1

Lymphoprep Axis Shield NYC1114545 L2

Fetal bovine serum Sera Lab Ltd 4/101/500/638 B3

1% Virkon solution BDH Merck 222/0154/06. V1

70% IMS Pharmacy N/A I9

1% acetic acid BDH 27013 A2

IL-2 (Proleukin)4.017 -80°C Chiron PROV1 I11

PHA 4.017 -80°C Bio stat HA16 P5

AntiCD3 (Pelicluster) -20°C CLB(Mast) CLB M-1654 H13

CD107a FITC BD Pharmingen 555800 A9

CD56 PE BD Pharmingen 345810 A9

CD8 PerCP BD Pharmingen 345774 A9

CD3 (APC) BD Pharmingen 345767 A9

Cell Wash BD Pharmingen 349524 C12

FACS Lyse BD Pharmingen 349202 F2

Cell Fix BD Pharmingen 340181 C11

FACS flow BD Pharmingen 342003 F1

**C. CONSUMABLES**

**Item Supplier Cat. No.**

Sterile Universals Greiner 201171

Sterile yellow tips Greiner 70.760.002

Sterile blue tips Greiner 877270

Sterile 5ml pipettes Sarstedt 1253.0001

Sterile pasteur pipettes Elkay 127P51110S

5ml capped culture tubes Marathon 2054

FACS tubes Marathon 2052

**D. QUALITY CONTROL**

**Internal:** A ‘normal’ control sample is tested in parallel to patient samples. Control results are logged and trends monitored.

**External:** No external scheme is available for these assays.

**E. METHOD**

**DAY 1:**

1. Take the lymphoprep, RPMI and FCS out of the fridge and allow to come to room temperature.
2. All procedures should be performed in the safety cabinet using aseptic technique as the cells are to be cultured.
3. Obtain a molecular worksheet for the patient (if one has not already been prepared for perforin/SAP). Fill in the patient and control details, and record the volume of blood received for patient and control.
4. Label three universals for the control and for the patient and place in the universal rack.
5. For each sample, pour the blood into one of the labelled universals. If perforin/SAP analysis is required, leave 200μl/assay in the collection tube.
6. Dilute the blood with an approximately equal volume of RPMI and mix gently.
7. Pour approx. 10ml lymphoprep into a second labelled universal. If the diluted blood volume is greater than 15ml, two lymphoprep tubes will be needed.
8. Swirl the lymphoprep round the universal to coat the sides.
9. Layer the diluted blood carefully onto the lymphoprep trying to minimise mixing.
10. Centrifuge at room temperature, 10min, 2500rpm, ensuring that the brake is off.
11. During this spin, if required, prepare 50ml of 10% FCS/RPMI by pipetting 45ml RPMI into a universal and adding 5ml FCS. Label the 50ml tube with ‘10% FCS/RPMI for GRA’, the date and your initials.
12. Place the spun samples in the universal rack and using a sterile pasteur pipette remove the mononuclear cells from the interface between the lymphoprep and the plasma/RPMI layers and place in the third labelled universal.
13. Top up each universal with RPMI, mix gently, and centrifuge at room temperature for 7min at 1500rpm with the brake on.
14. Tip off supernatant into a discard container containing Virkon and resuspend the cell pellet by flicking the bottom of the universal with a forefinger.
15. Resuspend the cells in 1ml of RPMI.
16. Obtain an aliquot of IL-2 (100,000 U/ml) from the -80°C freezer and allow to thaw. ***Note this IL-2 is a different concentration to IL-2 used for other assays in the lab***
17. A minimum of 2 x 10^6 cells is required for the GRA and the rest may be used for a lysate. If a lysate is not required, use all the cells for the GRA*.* Routinely, use all the cells for the GRA.
18. Calculate the volume of cell suspension required for 2x10^6^ cells and record on the molecular worksheet (Note; if lysates are not required all of the cells may be used):

Volume required (μl) = (2x10^6^/total number of cells) x 1000μl

1. Transfer the calculated volume of cell suspension into a sterile 5ml capped culture tube, labelled with ‘Control + IL-2’ or ‘[Patient name] + IL-2’, and add 10% FCS/RPMI to make a total volume of 4ml (4mls-ml of cells=media volume to add in mls)
2. Add 4 μl IL-2 (100,000U/ml), cap the tubes tightly and invert gently to mix.
3. Loosen the caps and incubate overnight at 37°C in CO_2_.
4. If Munc13-4/syntaxin-11/Munc 18-2 analysis is required, spin down the remaining cells (1500rpm, 7min, brake on) and prepare a cell lysate by following ISOP 045 Cell manipulation for Molecular Immunology. Use 20μl lysis buffer per 10^6^ cells to a maximum of 400μl.
5. Store the remaining 10% FCS/RPMI in the fridge overnight.

**DAY 2:**

1. Take the 10% FCS/RPMI and FITC-CD107a antibody out of the fridge. Obtain one aliquot of PHA (320μg/ml) and one aliquot of anti-CD3 antibody (7.5μg/ml) from the -20°C freezer and allow to thaw.
2. Remove the tubes from the incubator, close the caps tightly, and centrifuge in the immunofuge for 45 seconds on high speed.
3. Pour off the supernatant into a discard pot containing Virkon in the safety cabinet.
4. Break up the cell pellet by gently flicking the tube and resuspend the cells in 1.5ml 10% FCS/RPMI.
5. Label two capped 5ml culture tubes, one with ‘anti-CD3’ and the other with ‘PHA’, for the control and two for the patient.
6. Flick the cell suspension gently to make sure cells are well mixed, and then transfer 500μl of cell suspension into each of these two tubes, leaving the remainder in the original tube. This will be the ‘unstim’ tube.
7. Add 10μl anti-CD3 antibody to the ‘anti-CD3’ tubes.
8. Add 10μl PHA to the ‘PHA’ tubes.
9. Add 5μl FITC-antiCD107a antibody to the original tubes now labelled ‘unstim’, ‘anti-CD3’ and ‘PHA’ tubes.
10. Flick the tubes gently to mix, and incubate the tubes at 37°C in CO_2_ for 2h, making sure the caps are loose.
11. Remove the tubes from the incubator and discard the caps. The rest of the procedure can be done on the bench.
12. Spin the cells down in the immunofuge for 45 seconds at high speed, tip the supernatant down the sink with plenty of running water and blot the tubes on tissue.
13. Flick the tubes to resuspend the cell pellet.
14. Prepare antibody mix in a FACS tube: (no. of tubes +1) x 5μl PE antiCD56

(no. of tubes +1) x 5μl PerCP antiCD8

(no. of tubes +1) x 5μl APC antiCD3

(no. of tubes +1) x 35μl cell wash

| Number of tubes | antiCd56 PE | antiCD8 PCP | antiCD3 APC | Cell Wash |
| --- | --- | --- | --- | --- |
| 6 | 35 | 35 | 35 | 245 |
| 9 | 50 | 50 | 50 | 350 |
| 12 | 65 | 65 | 65 | 455 |
| 15 | 80 | 80 | 80 | 560 |

This mix should be made immediately before use and is to minimise pipetting.

1. Add 50μl antibody mix to each tube, flick to mix, and incubate for 10min at room temperature.
2. Add 1 ml FACS lyse, flick gently to mix, and incubate for 10min at room temperature. This ‘cleans’ the sample if there are lots of red blood cells (common in HLH patients).
3. Spin the cells down in the immunofuge for 45sec at high speed, tip the supernatant down the sink with plenty of running water and blot the tubes on tissue.
4. Flick the tubes to resuspend and add 1ml of cell wash.
5. Spin the cells down in the immunofuge for 45sec at high speed, tip the supernatant down the sink with plenty of running water and blot the tubes on tissue.
6. Flick the tubes to resuspend and add 250μl Cell Fix **(Toxic)**. Samples can be stored in the fridge overnight before analysis by flow cytometry, if covered in parafilm and wrapped in foil to protect from light. Tubes should not be left longer than 24h after staining before acquisition.

**Acquisition & Analysis**

1. Open the Granule Release Assay Acquisition template in the folder: Clinical Lab/Molecular Immunology/ /Granule release assay/ Granule acquisition.
2. Connect to the cytometer. Use GRA instrument settings (in the folder: Clinical Laboratory/Molecular Immunology/ Granule release assay)**.**
3. In the parameter settings, select the folder to save results in and name the file appropriately. For sample ID, type control or the patient’s hospital number and lab number. For patient ID, type ‘control for [patient’s initials]’ or the full patient name. Save in Molecular Immunology/year/month/day and write file path on the worksheet.
4. Acquire the tubes for each sample in the order ‘unstim’, ‘anti-CD3’, and ‘PHA’. If necessary, adjust R1 and R2. R2 is gating CD3+CD8+ cells and the template is set to acquire 10,000 R2 events. Change the file details between the control and the patient.
5. To analyse, open the analysis template: Clinical Lab/Molecular Immunology /Granule release assay/Granule analysis. Follow the steps below for each sample (control or patient).
6. Select the 1^st^ plot and open the first file (unstimulated). In the top left plot, check that the R1 gate is tightly around lymphocytes (FSC/SSC).
7. In the top middle plot, change the data file to the second file (anti-CD3).and check that the R2 gate is around the CD3+ CD8+ cells. Enlarge the gate if necessary to include all the CD3+CD8+ cells (the population shifts slightly to the left when stimulated).
8. In the top right plot change the data file to the third file (PHA) and check that the R3 gate is around the CD56+ CD3- cells. Adjust the gate if necessary.
9. In the first CD8 plot, change the data file to 0.001 (unstim), adjust the quadrant if necessary to have no more than 1% CD107a+ cells. In the second CD8 plot, open the second file (anti-CD3). If you adjusted the quadrant above, copy and paste onto this second plot, so that both plots have the same quadrants.
10. In the first NK plot, ensure data file unstim (0.001) is selected and adjust the quadrant if necessary to have no more than 5% CD107a+ cells (there is always some background staining). In the second NK plot, open the third file (PHA). If you adjusted the quadrant above, copy and paste onto this second plot, so that both plots have the same quadrants.
11. The percentage of CD107a+ cells (upper right quadrant for CD8+ T cells; lower right quadrant for NK cells) should be highlighted for each plot. The results are discussed at blot reading.
12. When entering the results into the Molecular Immunology Database, select ‘GRA (CD107a)’ under ‘Test requested’. Under results record, GRA normal, GRA abnormal, GRA absent. In the ‘Comments’ box, record the percentage of CD107a+ cells for CD8+ T cells and NK cells for the patient and the control.

**E. INTERPRETATION OF RESULTS**

Interpretation of results is performed at blot reading by two senior staff (senior clinical scientists or consultants).

An abnormal GRA result is when the increase in %CD107a between stimulated and unstimulated samples was <1.5% for cytotoxic T cells after anti CD3 stimulation and/or <15% for NK cells after PHA stimulation.

**CD8+ T cells:** There should be <1% CD107a+ cells when unstimulated. After anti-CD3 stimulation, there should be an increase in CD107a+ cells reaching up to 10^2^. In HLH with defective granule release, there will be no CD107a+ cells.

**NK cells:** Only analyse if there are more than 300 NK cells. There should be <5% CD107a+ cells when unstimulated, but there are always a few CD107a+ cells. After PHA stimulation, there should be >5% CD107a+ cells reaching up to 10^2^. In HLH with defective granule release, there will be no increase in CD107a+ cells above unstimulated.

**2. Detection of Perforin by FACS**

**A. EQUIPMENT**

Rack

P20 Gilson pipette

P200 Gilson pipette

Eppendorf Repeater

Immuofuge

FACSCalibur

B. REAGENTS

Reagent Supplier Cat. No. Hazard

Anti-perforinFITC BD 556577 A9

+ isotype FITC 2x1ml

Anti-CD56PE BD 345810 A9

1ml

Anti-CD4PerCP BD 34775 A9

1ml

Anti-CD8APC BD 34775 A9

1ml

Facs Lyse (Harmful) BD 349202 F2

1x1l

Cytofix/CytoPerm BD 554722 C20

(Harmful) 1x100ml

Perm/Wash Buffer BD 554723 RD7

(Toxic) (100 ml 10X stock, dilute 10 mls buffer -> 90 mls water)

Cell Fix BD 340181 C11

(Toxic) 1x500ml

Facs Flow BD 342003 F1

1x20l

**C. CONSUMABLES**

Item Supplier Cat. No.

Bench guard Marathon SA215-10

Facs tubes Marathon 2052

Yellow tips Griener 877290

12.5ml Distritips Anachem 12.5 Maxi

Tissue roll Marathon N/A

**D. QUALITY CONTROL**

Internal: A fresh ‘normal’ control is run weekly to check the settings.

Commercial beads are run weekly to check the instrument.

A ‘normal’ control sample is tested in parallel to patients samples

External: UK NEQAS scheme for Immunophenotyping.

**E. METHOD**

1. Place a piece of bench guard on the area of bench.
2. Label each tube with the patient’s initials and isotype or perforin. Using the table below add 5 uL of α-CD56, α-CD4, and α-CD8 to the bottom of the tubes being careful not to contaminate the tip with the other antibodies.

| Control | Control | Patient | Patient |
| --- | --- | --- | --- |
| Isotype Fitc | Anti-perforin Fitc | Isotype Fitc | Anti-perforin Fitc |
| α-CD56 | α-CD56 | α-CD56 | α-CD56 |
| α-CD4 | α-CD4 | α-CD4 | α-CD4 |
| α-CD8 | α-CD8 | α-CD8 | α-CD8 |

1. Add 100ul of whole blood to the bottom of the tube, taking care not to contaminate the tip with the antibody and not leaving blood on the sides of the tube where it cannot be mixed with the antibody. (If the patient is lymphopenic, 200 uL of blood may be added.)
2. Mix all the tubes well and incubate at room temperature for 10 minutes.
3. Add 1ml of Facs lyse (Harmful) to all the tubes, mix well and incubate at room temperature for 10 minutes.
4. Immunofuge on high for 45 secs, tip the supernatant down the sink with plenty of running water and blot the tubes on tissue.
5. Mix all the tubes to resuspend, add 250 uL Cytofix/Cytoperm (Harmful) solution and mix gently. Incubate for 20 minutes at room temperature.
6. Wash the cells twice with 1 ml 1X Perm/Wash buffer (Toxic) (Add 1 ml, tap, spin 45 seconds in the immunofuge on high, decant and blot on a tissue).
7. Resuspend the cells in the remaining volume of Perm/Wash (Toxic) post decant (~50 uL) by gentle mixing. Add 5 uL of anti-perforin antibody or the isotype control to the appropriate tubes, mix and incubate at room temperature for 10 minutes.
8. Wash the cells twice with perm/wash buffer (Toxic).
9. Resuspend in 250ul Cell Fix (Toxic).
10. Acquire 100,000 events using the CellQuest with 4 colour lyse wash instrument settings. Remove the secondary threshold to ‘none’. Use the perforin (Mol imm/perforin) acquisition template. Gate on lymphocytes. Remember that the forward and side scatter will have shifted due to the permeabilisation of the cells so the gate may have to be moved. Store the file under molecular immunology/patients/year/month/ new file with day’s date. Use C to prefix control files and the patient’s initials to prefix their files. See Facs acquisition SOP.
11. Analyze using the Facs analysis SOP and the perforin analysis template. Use overlayed histograms gating on the CD56+ cells, then the CD4+ cells and finally the CD8+ cells. Gate on all CD56+ cells but only on Cd4+ high and CD8+ high cells. Perforin is abnormal if <50% expression and/or fails to have the brightest cells reach Mean Fluorescein Intensity of 10^3^

**3.** **Detection of XIAP and SAP by FACS**

**A. EQUIPMENT**

Rack

P20 Gilson pipette

P200 Gilson pipette

Eppendorf Repeater

Immuofuge

FACSCalibur

**B. REAGENTS**

**Reagent Supplier Cat. No. Hazard**

Anti-mouse IgG1FITC DAKO A9

1x1ml

Anti-XIAP BD 610763 A9

100ul

Anti-SAP Abnova H00004068-M01

IgG1 isotype control BD 349040 A9

Anti-CD56/16 PE BD A9

Anti-CD8 PerCP BD A9

Anti-CD3 APC 4 BD A9

Facs Lyse BD 349202 F2

1x1l

Perm/Fix 1 x 1 ml Coulter

1x 30ml

SAP wash Lab solutions

1 x 500 ml

Cell Fix BD 340181 C11

1x500ml

Facs Flow 4.015 Bench/Floor BD 342003 F1

1x20l

SAP Block 4.015 Fridge Lab Solutions

**C. CONSUMABLES**

**Item Supplier Cat. No.**

Bench guard Marathon SA215-10

Facs tubes Marathon 2052

Yellow tips Griener 877290

12.5ml Distritips Anachem 12.5 Maxi

Tissue roll Marathon N/A

**D. QUALITY CONTROL**

**Internal:** A fresh ‘normal’ control is run weekly to check the settings.

Commercial beads are run weekly to check the instrument.

A ‘normal’ control sample is tested in parallel to patient’s samples

Genetic analysis may be performed on DNA from patients with absent/abnormal XIAP

**External:** UK NEQAS scheme for Immunophenotyping.

**E. METHOD**

1. Place a piece of bench guard on the area of bench.
2. Label each tube with the patient’s initials and isotype, SAP or XIAP. Add 50 ul of blood to the bottom of the tube.
3. Add 100 ul of solution 1 Perm/Fix and to each tune and mix immediately upon addition. Incubate at room temperature for 15 minutes.
4. Add 1 ml of SAP wash, mix, add a further 2 mls of SAP wash, spin 45 seconds in the immunofuge, decant and drain well.
5. Add 100 ul solution 2 Perm/Fix. Do NOT mix but let diffuse naturally into cell pellet. Incubate at room temperature for 5 minutes.
6. Disperse the cell pellet and add 1 uL of XIAP, 0.5 ul anti SAP or 6 ul of mouse isotype control antibody to the bottom of appropriate tubes. Incubate at room temperature for 15 minutes.
7. Wash 2 times with 2mls per wash of SAP wash.
8. Add 2.5 ul of anti-mouse IgG FITC and incubate for 15 minuets
9. Wash 2 times with 2mls SAP wash.
10. Add 5ul of CD56 PE, CD8 PerCP and CD3 APC to each tube. For ease this can be made as a premix (e.g. for 4 tubes combine 25 ul CD56 PE, CD8 PerCP and CD3 APC and pipette 15 ul into each tune). Incubate at room temperature for 10 minutes.
11. Wash 2 times with 1ml per wash of SAP wash.
12. Add 250 ul Cell fix.
13. Acquire 20,000 CD3+ events using the XIAP acquisition template (clinical lab/molecular immunology/R&D/XIAP). Ensure SAP instrument settings are used (molecular immunology/SAP). Store the file under molecular immunology/R&D/XIAP/ new file with day’s date. See Facs acquisition SOP.
14. Analyse using the Facs analysis SOP and the XIAP analysis template. SAP and XIAP are abnormal if <50% of cells express SAP/XIAP.

**Representative data of flow cytometry**
