# Supplementary material for: Screening assays for primary haemophagocytic lymphohistiocytosis in children presenting with suspected macrophage activation syndrome
Source: Pediatr Rheumatol Online J. 2015 Nov 16;13:48. doi: 10.1186/s12969-015-0043-7 (PMC4647814; doi:10.1186/s12969-015-0043-7)
Supplement: Supplementary file 2 [file 12969_2015_43_MOESM2_ESM.docx]

**Davi MAS sJIA Criteria**										
Number of Laboratory criteria	1	0	0	0	2	3	0	0	1	2
Plt < 262 x 10^9/l	yes	no	no	no	yes	yes	no	no	**yes**	yes
AST > 59 IU/l	/	/	/	/	/	yes	/	/	/	yes
WCC < 4 x 10^9/l	no	no	no	no	yes	yes	no	no	no	no
Number of clinical features	2	1	1	2	2	2	0	1	1	1
CNS dsyfunction - defined as irritability, disorientation, headache, seizure or coma	yes	yes	no	yes	yes	yes	no	no	no	no
Haemorrhages	no	/	/	/	/	/	/	/	/	/
Hepatomegaly	yes	no	**yes**	yes	yes	yes	no	**yes**	**yes**	yes
**SoJIA MAS criteria: ≥ 2 lab criteria, or ≥ 2 clinical and/or laboratory criteria**	yes	no	no	yes	yes	yes	no	yes	yes	yes
Ferritin > 500ng/ml	no	no	**yes**	yes	yes	yes	**yes**	**yes**	**yes**	yes
Histopathogical criteria Bone Marrow Aspirate evidence of haemophagocytic lymphohistocytosis	no	no	no	no	/	no	**yes**	yes	yes	no
**Other clinical info**										
Arthritis	yes	no	**yes**	no	no	**yes**	**yes**	**yes**	**yes**	**no**
Ferritin ng/ml	440	121	21065	1679	523	6759	2801	22076	17800	2050
ALT IU/L	21	11	17	17	12	453	6	354	227	125
ESR mm/hr	9	24	100	10	78	20	18	120	4	9
CRP mg/L	62	<5	48	74	60	/	51	87	38	29
**Screening Tests**										
GRA	unable to interpret	unable to interpret	**abnormal**	**abnormal**	**abnormal**	**abnormal**	**abnormal**	**abnormal**	normal	**/**
SAP	/	normal	/	/	/	normal	/	**/**	/	normal
XIAP	/	/	/	/	/	normal	**/**	**/**	**/**	**/**
PERFORIN	normal	normal	normal	normal	normal	normal	/	normal	normal	normal
